# New Palladium(II) Complexes Containing Methyl Gallate and Octyl Gallate: Effect against *Mycobacterium tuberculosis* and *Campylobacter jejuni*

**DOI:** 10.3390/molecules28093887

**Published:** 2023-05-05

**Authors:** Raphael Tristão Cruvinel Silva, Micaela Guidotti-Takeuchi, Jéssica Laura Miranda Peixoto, Fernanda Manaia Demarqui, Ananda Paula Mori, Carolyne Ferreira Dumont, Gabriella Rayane Aparecida Ferreira, Gabriele de Menezes Pereira, Daise Aparecida Rossi, Pedro Paulo Corbi, Fernando Rogério Pavan, Celso de Oliveira Rezende Júnior, Roberta Torres de Melo, Wendell Guerra

**Affiliations:** 1 Institute of Chemistry, Federal University of Uberlândia—UFU, Santa Mônica Campus, Uberlândia 38402-018, MG, Brazil; tristao@ufu.br (R.T.C.S.);; 2 Laboratory of Experimental Molecular Epidemiology, Federal University of Uberlândia—UFU, Umuarama Campus, Uberlândia 87504-000, MG, Brazil; micaela.guidottitakeuchi@ufu.br (M.G.-T.);; 3 Faculty of Pharmaceutical Sciences, Paulista State University—UNESP, Araraquara Campus, Araraquara 14800-060, SP, Brazil; 4 Institute of Chemistry, State University of Campinas—UNICAMP, Campinas 13083-872, SP, Brazil

**Keywords:** palladium(II) complexes, antibacterial activity, anti-infective drugs, *Mycobacterium tuberculosis*, *Campylobacter jejuni*

## Abstract

This work describes the preparation, characterization and antimicrobial activity of four palladium(II) complexes, namely, [Pd(meg)(1,10-phen)] **1**, [Pd(meg)(PPh_3_)_2_] **2,** [Pd(og)(1,10-phen)] **3** and [Pd(og)(PPh_3_)_2_] **4**, where meg = methyl gallate, og = octyl gallate, 1,10-phen = 1,10-phenanthroline and PPh_3_ = triphenylphosphine. As to the chemical structures, spectral and physicochemical studies of **1**–**4** indicated that methyl or octyl gallate coordinates a palladium(II) ion through two oxygen atoms upon deprotonation. A chelating bidentate phenanthroline or two triphenylphosphine molecules complete the coordination sphere of palladium(II) ion, depending on the complex. The metal complexes were tested against the *Mycobacterium tuberculosis* H37Rv strain and **2** exhibited high activity (MIC = 3.28 μg/mL). As to the tests with *Campylobacter jejuni*, complex **1** showed a significant effect in reducing bacterial population (greater than 7 log CFU) in planktonic forms, as well as in the biomass intensity (IBF: 0.87) when compared to peracetic acid (IBF: 1.11) at a concentration of 400 μg/mL. The effect provided by these complexes has specificity according to the target microorganism and represent a promising alternative for the control of microorganisms of public health importance.

## 1. Introduction

The design and synthesis of metal-based drugs has been a vigorous area of research since the introduction of cisplatin as the first platinum drug applied for cancer treatment in the 1970s [1,2]. After cisplatin, two platinum-based complexes were approved as antitumoral drugs worldwide, namely, carboplatin and oxaloplatin, while five other platinum drugs were approved in individual countries [3]. Platinum drugs have been a key component of cancer chemotherapy; for example, cisplatin is involved in the treatment of different types of cancer, including sarcomas, cancers of soft tissue, bones, muscles, and blood vessels [4]. In addition to platinum drugs, several non-platinum metal complexes are also available for medicinal applications, such as silver, bismuth, and gold complexes as antimicrobial, antiulcer and anti-rheumatic agents, respectively. The effectiveness of bismuth complexes has been attributed to their bactericidal action against the bacterium *Helicobacter pylori*, and silver compounds have been known as bacterial agents since ancient times [5,6]. There are many potential applications for metal-based drugs, but without a doubt, the emergence of bacterial resistance, with consequent decrease in the arsenal of drugs currently available make the metal compounds a promising source for new antibiotics that should be explored [7,8]. Therefore, the synthesis of new metal-based drugs as antimycobacterial, antifungal and antibacterial agents is highly desirable. 

Palladium(II) has a coordination chemistry very similar to that of platinum(II), and various palladium(II) complexes have been proposed as drug candidates due to their excellent antitumor and anti-infective activities in vitro and in vivo [5]. One of the most recent examples is padeliporfin (Tookad^®^Soluble), a water-soluble palladium complex used in vascular targeted photochemotherapy (VTP) for the treatment of low-risk prostate cancer [9]. Palladium(II) complexes containing the isonicotinamide ligand have shown higher activity against *Mycobacterium tuberculosis* than free isonicotinamide and pyrazinamide [10]. Additionally, palladium(II) complexes containing thiosemicarbazones have been evaluated against *Staphylococcus aureus*, *Streptococcus pyogenes*, *Salmonella typhimurium*, and *Escherichia coli* and showed good antibacterial activity when compared with antibiotic amoxicillin [11]. In addition to the complexes mentioned above, palladium(II) complexes bearing fluoroquinolones antibiotics have been evaluated against *Mycobacterium tuberculosis* virulent strain H37Rv. The complexes containing sparfloxacin were the most active, and inhibited bacterial growth at 0.31 μg/mL. It was also observed that all complexes, except the palladium complex with ciprofloxacin, were more active than rifampicin, a first-line oral anti-tuberculosis drug [12]. Palladium(II) complexes containing tetracycline antibiotics were synthesized by our group and tested against bacterial strains sensitive and resistant to tetracycline. The palladium(II) complex with tetracycline was 16 times more potent than free tetracycline against the resistant bacterial strain (*Escherichia coli* HB101/pBR322). It was also verified that the palladium(II) complex with doxycycline was two-fold more active in the resistant strain than free doxycycline [13].

Gallic acid and its ester derivatives present many biological activities, such as antioxidant, anticarcinogenic, antimicrobial, antifungal, antimutagenic, antiangiogenic, anti-inflammatory, and are widely used in the food and pharmaceutical industries [14,15,16,17,18,19,20,21,22]. Choi et al. evaluated the antibacterial activity of methyl gallate (meg) against various *Salmonella* strains and the MIC values ranged from 3.9 to 125 μg/mL. It was also observed that meg treatment significantly increased the survival of animals from *Salmonella* infection, whist in untreated groups all animal succumbed to disease after a few days post infection, which revealed the therapeutic capacity of meg against *Salmonella* infections [23]. Shi et al. reported that octyl gallate (og) showed a remarkable antibacterial activity against *Escherichia coli* and *Staphylococcus aureus* when compared to other alkyl gallates, via a multiple bactericidal mechanism [24]. 

These reports motivate us to use alkyl gallates as ligands for the synthesis of metal complexes as potential new anti-infective agents. Gallic acid and its ester derivatives can coordinate metal ions via two oxygen atoms in a bidentate manner, and the resulting metal complexes can exert interesting biological properties [25]. For instance, two metal complexes were prepared with propyl gallate (PG), namely, [Pt(PG)(PPh_3_)_2_] and [Ru(PG)(dppm)_2_], where dppm = 1,1-bis(diphenylphosphino) methane. The cytotoxicity of the complexes against four tumor cell lines was evaluated and the results showed that the ruthenium complex was potent and selective while the platinum complex was inactive. However, it was observed that only [Pt(PG)(PPh_3_)_2_] complex presents an inhibitory effect against free radicals [26]. Graminha et al. reported that ruthenium(II) complexes with gallic acid and derivatives exhibited high cytotoxic activity against MDA-MB-231 cells. It was also observed that ruthenium complex with gallic acid was able to cause damage to the cellular cytoskeleton leading to inhibition of some cellular processes of MDA-MB-231 cells, such as invasion, migration, and adhesion [27]. Other studies have also indicated that platinum complexes containing gallic acid analogues are very promising as anticancer agents [28,29].

Promising compounds are also being widely investigated for the control of pathogens of high public health importance [30]. Campylobacteriosis is the leading microorganism of gastrointestinal diseases in humans caused by bacteria in the world. In the US, 25,866 cases of *Campylobacter* bacterial infection have been reported [31,32] and in the European Union, with expressive 120,946 cases reported in 2020 [33]. However, in Brazil, bacteria of the genus *Campylobacter* were not part of the results of the investigation of pathogens that caused more diarrhea between 2010 and 2018 in the population [34], yet several investigations reaffirm the problems of biofilm formation and multi-drug antimicrobial resistance concerning this pathogen in the country [35,36,37,38,39,40].

Considering the above discussions, our objective was to prepare new palladium(II) complexes containing meg and og and evaluated them against *Mycobacterium tuberculosis* and *Campylobacter jejuni* strains, a Gram-negative bacterium responsible for enteric infection in humans, in planktonic and sessile forms.

## 2. Results and Discussion

### 2.1. Synthesis and Spectroscopic Characterization of Complexes **1**–**4**

**Figure 1:**
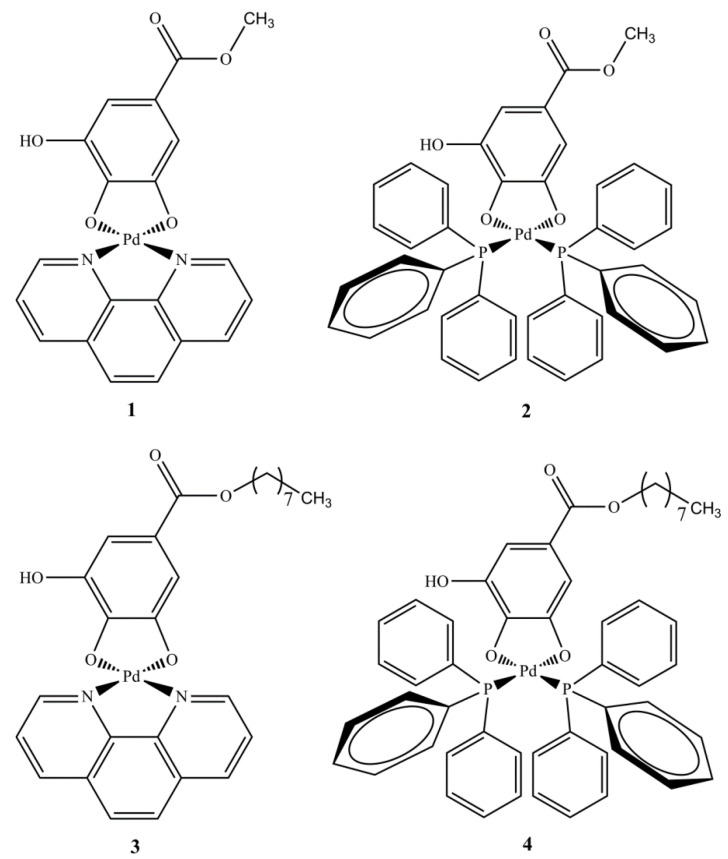
Proposed structures for the palladium(II) complexes **1**–**4**.

This work reports, as described in the Experimental Section, the synthesis of four palladium(II) complexes containing two gallic acid derivatives as ligands. The complexes were prepared in good yields under mild conditions. All complexes are colorful solids, stable to air and light and soluble in DMSO and DMF. In their structures, gallic acid derivatives act as dianionic ligands, coordinating with the palladium via two oxygen atoms, as previously reported in the literature [26,27]. Additionally, a phenanthroline or two triphenylphosphine molecules acting as a monodentate ligands complete the coordination sphere of palladium(II) ion, depending on the complex (see Figure 1). According to the studies performed, the complexes were prepared with high purity and are non-electrolytes [2]. The mass spectra of complexes **1**–**4** were recorded, and the results were in agreement with their proposed structures. For example, the mass spectrum of **1** showed a charged complex ion at *m*/*z* 469.0009 [M + H]^+^, according to the calculated value for [Pd(C_8_H_6_O_5_)(C_12_H_8_N_2_)+H]^+^, 469.0010 (Δ −0.2132 ppm).

**Figure 2:**
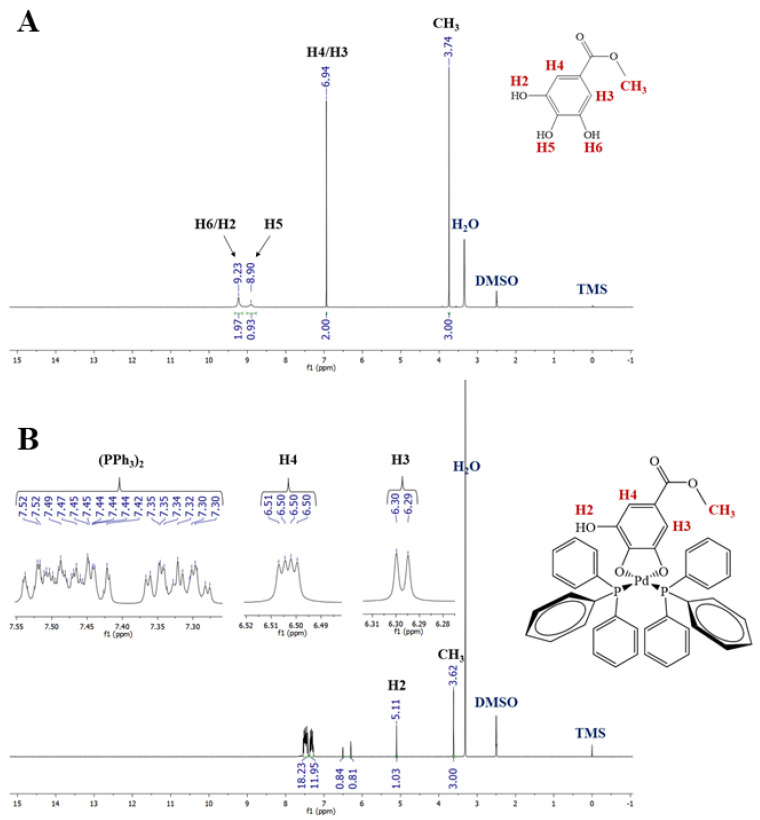
^1^H NMR spectrum of methyl gallate (**A**). ^1^H NMR spectrum of complex **2** (**B**).

The ^1^H NMR spectrum of meg ligand (Figure 2A) in DMSO-*d_6_* showed signals at 3.74 (CH_3_ group), 6.94 (H3/H4), 9.90(H5) and 9.23 (H2 and H6) ppm. In the ^1^H NMR spectra of complexes **1** and **2** (see Supplementary Materials Figure S2 and Figure 2B), the signals corresponding to the CH_3_ group and the H3/H4 protons were less affected than H2 when compared with the related groups of free meg. The absence of H5 and H6 suggests the participation of meta and para oxygen atoms in the coordination. The ^13^C{^1^H} NMR spectra of these complexes (see Supplementary Materials Figures S7 and S8) showed a signal close to δ 166.25 (δ 167.23 and 167.22 for complexes **1** and **2**, respectively), which was attributable to carbon C=O. This signal was slightly affected by coordination, when compared to the signal from the free meg (see Supplementary Materials Figure S5), which excluded the involvement of C=O group in the coordination to the metal. On the other hand, according to the results of the ^1^H NMR spectroscopic experiments, the signals corresponding to carbons C5 and C6 were the most affected, which reinforces the participation of the meta- and para-hydroxyl OH groups in the coordination to metal. The changes observed in the ^1^H and ^13^C{^1^H} NMR spectra of the complexes (see Supplementary Materials Figures S3, S4, S9 and S10) with og ligand (see Supplementary Materials Figure S1 and S6) were very similar and will not be discussed here. The ^31^P{^1^H} NMR spectra of complexes **2** and **4** exhibited two doublets, which indicates the presence of two non-equivalent phosphorus atoms in both complexes (see Supplementary Materials Figures S11 and S12) [26]. As to the IR spectra, meg and og showed two bands at ~3450 and 3300, which are typical of the phenol group [26]. A band at 1688 cm^−1^ can be assigned to carbonyl stretching ν(C=O) [41]. Regarding the infrared spectra of the complexes, the main observed change concerns the absorption of OH groups. For example, the OH band close to 3300 cm^−1^ disappeared in the spectrum of complexes **2** and **4** (See Supplementary Materials Figures S14 and S16), also suggesting the participation of OH groups in the coordination. On the other hand, in the IR spectra of complexes **1** and **3** (See Supplementary Materials Figures S13 and S15), a broad band at 3400–3550 cm^−1^ suggests the presence of water. In this sense, the TG curve of **1** was performed (see Supplementary Materials Figure S17), and a weight loss event in the range of 25 to 102°C is due to the loss of two water molecules (calculated: 7.14%; experimental: 7.80%). At 900 °C there is a residue (elemental palladium) corresponding to 20.81% (calcd: 21.08%).

### 2.2. Antimycobacterial Activity

Tuberculosis (TB) is an illness that affects about 10 million people worldwide, of whom approximately 1.5 million die from the disease each year. TB can be treated by taking various drugs, such as isoniazid and rifampicin over the course of a treatment lasting 6–9 months [42,43]. However, multidrug-resistant TB (MDR-TB) is a serious public health crisis, and makes the design and development of new drugs paramount. 

In this context, we evaluated the antimycobacterial activity of complexes **1**–**4**, as well as their ligands and control drugs, against the MTB H37Rv strain using the REMA method. The MICs of these compounds are shown in Table 1. Regarding organic compounds, meg was inactive at 25 μg/mL (MIC > 25 μg/mL), while og showed low activity (MIC 22.93 μg/mL). However, both complexes bearing meg were active against the MTB H37Rv strain, especially complex **2**, which exhibited an MIC value of 3.28 μg/mL. More specifically, this complex was approximately 3.45 times more active than **1** (MIC 11.31 μg/mL), which indicates that the replacement of a phenanthroline molecule by two triphenylphosphine groups enhances the antitubercular activity of these complexes. The same behavior was observed for complexes **3** and **4** (MICs > 25 μg/mL vs. 9.06 μg /mL, respectively). Considering the gallic acid ester derivatives, it was possible to verify that the complexes containing meg (complex **1** more active than **3** and complex **2** more active than **4**) were the most active, although og (MIC = 22.93 μg /mL) was more active than meg (MIC > 25 μg /mL). For example, complex **2** was about 2.76 times more active than complex **4** (MICs 3.28 μg/mL vs. 9.06 μg/mL, respectively). Finally, complexes **1**, **2** and **4** showed similar or superior activity when compared to other palladium(II) complexes described in the literature [10,44,45,46,47].

### 2.3. Selectivity against Campylobacter jejuni

**Figure 3:**
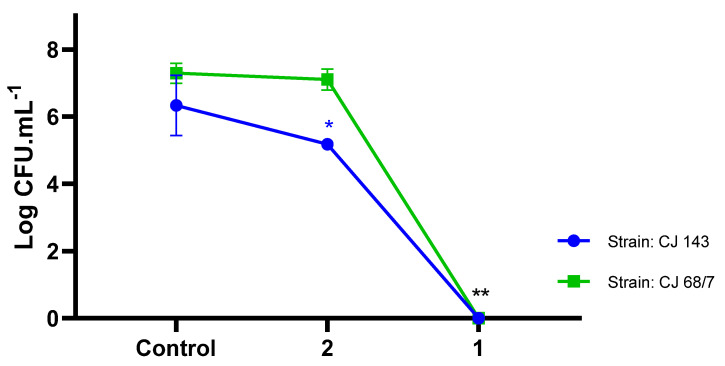
Reduced microbial counts of *Campylobacter jejuni* exposed to different palladium (400 μg/mL) metal compounds (**1** and **2**). CJ 143 and 68/7: *C. jejuni* strains. * *p* < 0.05, ** *p* < 0.0001 in one− way ANOVA test.

The control of antibiotic-resistant strains of *Campylobacter jejuni* is a challenge. The effects of compounds **1** and **2** were evaluated for the log reduction of the *Campylobacter jejuni* population (CJ143 and CJ68/7 strains) at a concentration of 400 μg/mL. Complex **1** showed a significant effect compared to **2** (*p* < 0.0001) at reducing the bacterial population (control group mean: 7.29 log CFU, and complex **1**: no growth considering the detection limit of the test; control group mean: 6.33 log CFU and complex **2**: 5.17 log CFU) at the highest concentration (400 μg/mL), showing high selectivity of action against *Campylobacter jejuni*. In free cells, compound **1** was shown to be a biologically active complex with sufficient action in both doses/concentrations evaluated, activating the processes enablign the control of the pathogen (Figure 3). 

When evaluating the lowest concentration (well H), represented by a concentration of 3.125 μg/mL, there was a reduction of 2.5 log CFU and 1.2 log CFU in complexes **1** and **2**, respectively (control group mean: 6.3 ± 0.9; complex **1**: 3.8 ± 0.4 log CFU and control group mean: 6.3 ± 0.9; complex **2**: 5.2 ± 0.2 log CFU). A recent report with *Pseudomonas aeruginosa* evaluated Pd coordination complexes with respect to toxicity and growth control of microorganisms, showing that different palladium complexes (sodium tetrachloropalladate (Na_2_[PdCl_4_]), tetraamminepalladium(II) chloride ([Pd(NH_3_)_4_]Cl_2_), and potassium hexachloropalladate(IV) (K_2_[PdCl_6_])) assist in the mitigation of wild-type pathogens and that the inhibitory capacity is dose dependent [48].

Even at lower concentrations (3.125 μg/mL), and facing the challenge of being antibiotic-resistant microorganisms, complexes **1** and **2** promoted reductions in the *Campylobacter jejuni* inoculum by 99% and 90%, respectively, representing MIC_99_ and MIC_90_. These results show that the quantitative reduction in logarithmic cycles at the lowest concentration evidences the effectiveness of the compound, as well as its applicability in sanitization processes. Moreover, it is a critical point to be considered, because it relieves the costs related to production under low concentrations.

The formulation of these four compounds is new; despite some palladium complexes having been evaluated for their ability to suppress bacterial growth, no results have been obtained from tests in *Campylobacter jejuni* for similar complexes [49,50,51].

All complexes were coordinated to a gallic acid ester derivative; however, the structure of complex **1** should be emphasized in comparison to different palladium(II) complexes previously tested, which showed results with Gram-negative microorganisms [52], and highlighted the role of methyl gallate with antimicrobial activity in *Salmonella*, demonstrating that the primary mechanism of action of meg was not through inhibition of cell wall synthesis, but rather through inhibition of DNA gyrase or ATPase in this pathogen [23]. 

### 2.4. Impact of Palladium Complexes on the Biomass of Campylobacter jejuni

**Figure 4:**
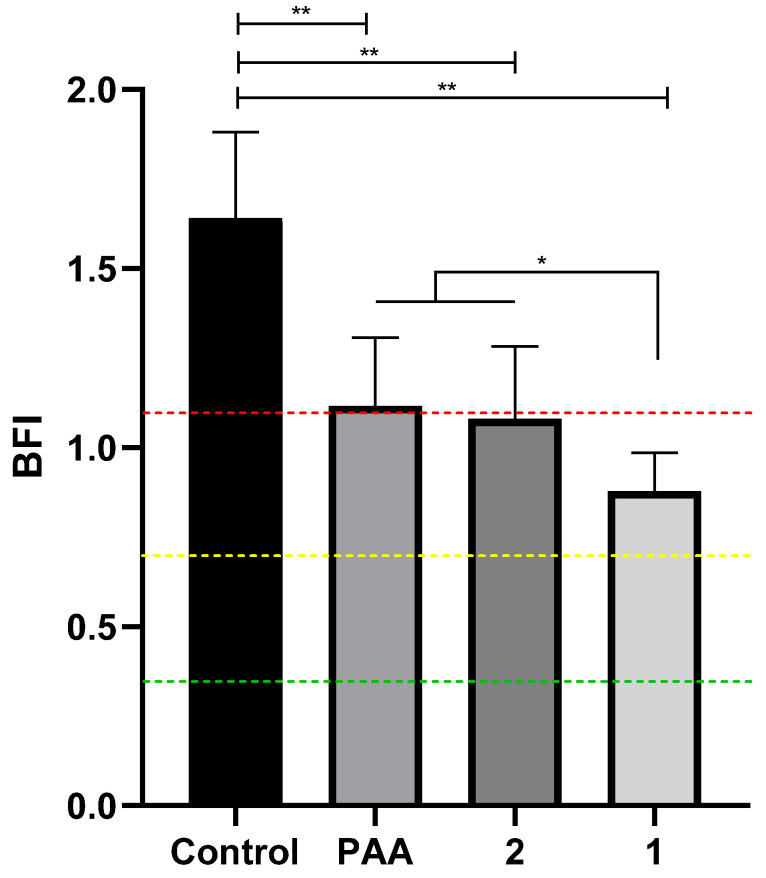
Effect of 800 μg/mL peracetic acid and treatment with different palladium complexes (**1** and **2**) (400 μg/mL) on biofilms supplemented with chicken juice from two strains of *C. jejuni*. The results represent means with standard deviation (error bars) of three independent experiments with eight replicates. Color lines indicate BFI classification limits; * *p* < 0.05; ** *p* < 0.001 using one way ANOVA.

When evaluating the qualitative biomass, the same concentration tested in planktonic cells (400 μg/mL) was used to compare the impact on sessile cells in order to develop a single-dose complex that could act efficiently in both forms (Figure 4). Biomass analysis for the control group in the presence of the *chicken juice* substrate qualified the strains as strong biofilm formers (BFI 1.63) [30,39].

After treatment with the standard commercial sanitizer (peracetic acid (PAA), 800 μg/mL) and palladium complexes, we observed a significant reduction in biomass intensity and reclassification to medium biofilm formation for all three treatments (IBF: 1.11; 1.08 and 0.87 to APA, complex **2** and **1**, respectively) compared to the control group (*p* < 0.0001), even in the presence of the *chicken juice* supplement.

The presence of *chicken juice* maximizes microbial growth [53], which characterizes evaluation using *Campylobacter jejuni* as a control in more difficult situations, such as the testing antimicrobial-resistant strains, and in conditions that mimic those in the slaughterhouse industry [54,55]. In addition, it was observed that multi-antimicrobial-resistant strains are strong biofilm formers and therefore require high complexity for their control in the food chain [56]. The use of metallic palladium compounds demonstrates potential for the reduction of biofilm cells. The use of palladium complexes alone [57] or in association [58] has also been shown to be a promising strategy against biofilms of *A. baumannii* and *Escherichia coli* J53, respectively. Thiazolinyl-picolinamide-based palladium(II) complexes showed good biofilm-breaking properties and intense inhibitory activity against standard and clinical strains of *Acinetobacter baumannii* in fighting implant infections [57]. Even in the face of the factors mentioned above, complex **1** showed the lowest IBF when compared with PAA at twice the concentration (800 μg/mL). BFI was reduced by 1.47, 1.51 and 1.87 orders of magnitude, respectively, for PAA, complex **2** and complex **1** compared to the control group.

Other experiments with palladium complexes have shown promising results on Gram-positive microorganisms and pathogens of importance for public health [59]. Concentration also played a key role in increasing the antimicrobial activity of the palladium under study against planktonic bacteria and biofilms in an environment that allowed microbial growth, contributing to studies maximizing the challenges encountered in practice [60].

**Figure 5:**
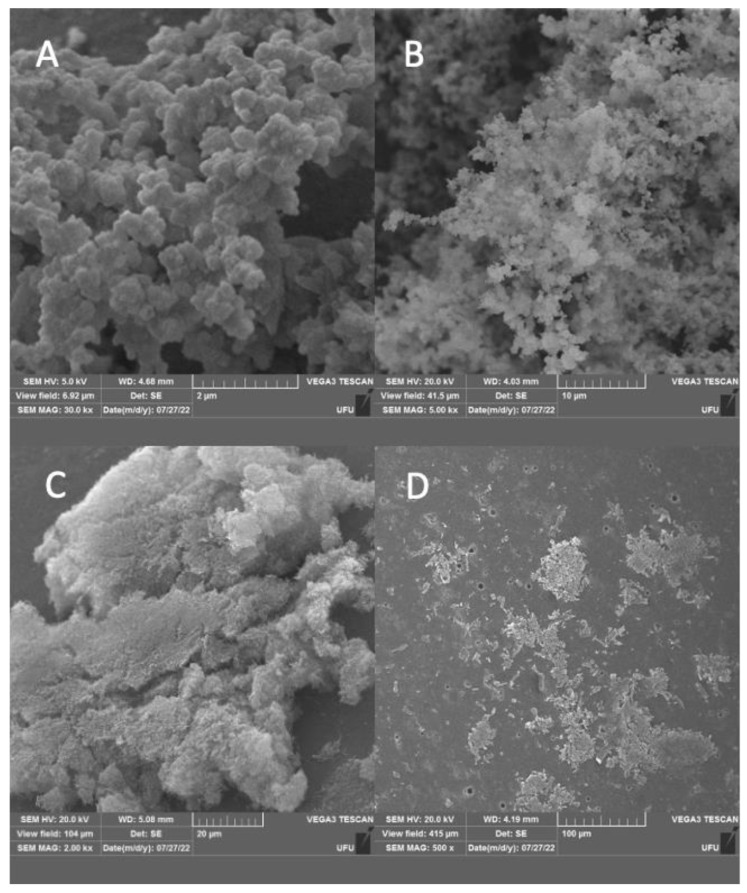
SEM images demonstrating the ultrastructure of the biofilm formation of *Campylobacter jejuni* in the control group (**A**), and with the use of the sanitizer PAA (**B**) and palladium complexes **1** (**D**) and **2** (**C**).

In the SEM assay, we observed modifications in the biomass formed for the three treatments applied at the initial stage of biofilm formation (PAA and complexes **1** and **2**). Figure 5A shows the presence of control group, indicating abundant biomass formation in the presence of organic matter, with an expanded and well-defined matrix. In Figure 5B, it can be observed that the biofilm in the presence of PAA exhibits three-dimensional structure with an evident and slightly more compacted matrix compared to the control group. The third morphology, complex **2** (Figure 5C), in which a reduction from a strong formation to an IBF of medium formation can be observed—0.7–1.10—we identified a moderately abundant biomass, with distinct characteristics of the matrix with compacted appearance. The most intense effect can be observed for complex **1** (Figure 5D), where intense fragmentation and destruction of the matrix is displayed. In addition, destabilization and flattening of the three-dimensional structure can be observed (Figure 5D). The intensity of the action of complex **1** on biofilms corroborates the results in sessile cells, and allows us to infer that complex **1** caused greater difficulty for biomass structuring at advanced stages of cell maturity. Unlike other pathogens, *Campylobacter jejuni* biofilms are necessary to protect the microaerophilic planktonic cells that can be both resistant and demanding, and therefore, a strategy interfering with these two forms represents a breakthrough with respect to control in biofilm formation [61].

Thus, the action of complex **1** in *C. jejuni* acts jointly on the free cells and inhibits the formation of mature biofilms by preventing the production of a homogeneous and stable structure.

## 3. Materials and Methods

### 3.1. Experimental Section

#### 3.1.1. Starting Materials

Bis(triphenylphosphine)palladium(II) dichloride or [PdCl_2_(PPh_3_)_2_] (98% pure), methyl gallate (meg) (98% pure), and octyl gallate (og) (≥97% pure) were purchased from Merck. In turn, [PdCl_2_(1,10-phen)] was prepared according to the procedure described in the literature [62]. All other chemicals (≥95% pure) were purchased from J.T Backer or Merck and were used as received without further purification. 

#### 3.1.2. Physical Measurements

Elemental analyses (% C, H and N) were performed using a Perkin Elmer 2400 CHNS/O Analyzer. Infrared spectra (4000–220 cm^−1^) were acquired on a PerkinElmer Frontier MIR spectrometer equipped with an attenuated total reflectance (ATR) sample holder with a diamond crystal. Conductivity measurements were performed on a Tecnopon mCA-150 instrument with a cell constant equal to 0.9585 cm^−1^ and spectroscopic grade dimethylsulfoxide (DMSO) as solvent (ɅM = 0.99 S cm^2^ mol^−1^).

High-resolution mass spectra (HRMS) of the complexes were obtained using an Orbi-trap Thermo Q-Exactive equipment. The electrospray ionization method (ESI) was used, and samples were prepared in a 1:1 acetonitrile: water solution containing 0.1% of formic acid. The solutions of the complexes were infused directly into the instrument’s ESI source. Samples were analyzed in the positive mode with flow rate of 200 μL min^−1^ and cone voltage of 3.5 kV in the range from 100 to 1500 *m*/*z*. 

The ^1^H NMR (400 MHz), ^13^C{^1^H} NMR (100 MHz), and ^31^P{^1^H} NMR (162 MHz) spectra were recorded on a Bruker AscendTM 400 Avance III HD spectrometer and were obtained after dissolving the complexes in DMSO-*d_6_*. The chemical shifts were expressed as δ (in ppm) from internal reference standard TMS (^1^H and ^13^C{^1^H} NMR).

Thermogravimetric analyses (TGA/DTA) of **1** were performed using a TGA-50 Shimadzu, with 7.254 mg of sample packed in an alumina crucible. The sample was heated at 10 °C/min from room temperature to 900 °C, in a dynamic air atmosphere (flow rate of 50 mL/min). 

#### 3.1.3. Synthesis of the Complexes **1**–**4**

To a methanolic suspension (5 mL) of 0.125 mmol (0.0877 g) of *trans*-[PdCl_2_(PPh_3_)_2_] or 0.125 mol (0.04468 g) of [PdCl_2_(phen)], depending on the complex, was added dropwise an equimolar amount of ligand (0.0256 g of meg or 0.0353 g of og) previously dissolved in 5 mL of methanol. Thereafter, six drops of triethylamine were added, and the reaction was stirred at room temperature for 48 h. The solids obtained were filtered off and washed with small amounts of methanol and dried under reduced pressure.

[Pd(meg)(1,10-phen)]·2H_2_O **1**

Yield: 92.36%. Color: Brownish red. Molar Weight (g mol^−1^): 504.7861. Anal. Calcd. for [Pd(C_8_H_6_O_5_)(C_12_H_8_N_2_)]·2H_2_O: C, 47.59; H, 3.59; N, 5.55%; Found: C, 47.24; H, 3.54; N, 5.76%. HRMS *m/z* 469.0009 (Calculated for [Pd(C_8_H_6_O_5_)(C_12_H_8_N_2_)+H]^+^ 469.0010 (Δ −0.2132 ppm)). ^1^H NMR (400 MHz; DMSO-*d_6_*) δ (ppm): 3.70 (s, 3H, CH_3_), 6.57 (d, *J* = 2.0 Hz, 1H, H3), 6.59 (d, *J* = 2.0 Hz, 1H, H4), 7.76 (s, 1H, H2), 8.08 (m, 2H, phen), 8.23 (s, 2H, phen), 8.88 (m, 3H, phen), 9.16 (dd, *J* = 5.10, 1.30 Hz, 1H, phen). ^13^C {^1^H} NMR (100 MHz; DMSO-*d_6_*) δ (ppm): 50.90 (C1), 104.94 (C2), 108.56 (C3), 115.52 (C4), 125.71, 125.94, 127.24, 127.44, 129.75, 129.80, 130.30, 138.81, 138.86, 139.88, 144.26, 145.48, 145.55, 149.55, 149.67 (phen), 150.02 (C5), 155.65 (C6), 162.68 (C7), 167.23 (C=O). FT-IR spectra in ATR, ν (cm^−1^): 3566, 3065, 2946, 1699, 1641, 1608, 1568, 1519, 1488, 1433, 1412, 1342, 1274, 1204, 1170, 1079, 1067, 1002, 912, 883, 850, 843, 812, 758, 727, 713, 598, 560, 516, 506, 488, 442, 417, 299, 227. Λ_M_ (10^−3^ M in DMSO) = 0.17 S cm^2^ mol^−1^.

[Pd(meg)(PPh_3_)_2_] **2**

Yield: 83.95%. Color: Grayish purple. Molar Weight (g mol^−1^): 813.1345. Anal. Calcd. for [Pd(C_8_H_6_O_5_)(C_18_H_15_P)_2_]: C, 64.99; H, 4.46%. Found: C, 64.54; H, 4.46%. HRMS *m*/*z* 813.1169 (Calculated for [Pd(C_8_H_6_O_5_)(C_18_H_15_P)_2_+H]^+^ 813.1146 (Δ +2.8286 ppm)). ^1^H NMR (400 MHz; DMSO-*d_6_*)) δ (ppm): 3.62 (s, 3H, CH_3_), 5.11 (s, 1H, H2), 6.30 (d, *J* = 2.0 Hz, 1H, H3), 6.50 (dd, *J* = 2.0, 1.0 Hz 1H, H4), 7.28–7.37 (m, 12H, PPh_3_), 7.42–7.54 (m, 18H, PPh_3_). ^13^C {^1^H} NMR (100 MHz; DMSO-*d_6_*) δ (ppm): 50.80 (C1), 102.89 (C2), 109.26 (C3), 115.37 (C4), 127.44, 127.97, 128.20, 128.26, 128.29, 128.39, 128.62, 128.73, 131.24, 131.35, 134.02, 134.14, 134.26 (PPh_3_), 143.63 (C5), 154.83 (C6), 163.07(C7), 167.72 (C=O). ^31^P{^1^H} (162 MHz; DMSO-*d_6_*) δ (ppm): 29.73 (d, *J* = 31.59 Hz, P1), 32.24 (d, *J* = 31.59 Hz, P2). FT-IR spectra in ATR, ν (cm^−1^): 3477, 3071, 3047, 2988, 2935, 1690, 1566, 1492, 1484, 1436, 1343, 1282, 1205, 1175, 1161, 1094, 1062, 1026, 1005, 911, 868, 814, 770, 744, 705, 689, 618, 585, 543, 517, 493, 469, 457, 442, 413, 359, 273. Λ_M_ (10^−3^ M in DMSO) = 0.53 S cm^2^ mol^−1^.

[Pd(og)(1,10-phen)]·2.5H_2_O **3**

Yield: 80.50%. Color: Brownish red. Molar Weight (g mol^−1^): 611.9722. Anal. Calcd. for [Pd(C_15_H_20_O_5_)(C_12_H_8_N_2_)]2.5H_2_O: C, 52.99; H, 5.44; N 4.58%. Found: C, 52.48; H, 4.74; N, 5.38%. HRMS *m/z* 567.1116 (Calculated for [Pd(C_15_H_20_O_5_)(C_12_H_8_N_2_)+H]^+^ 567.1106 (Δ +1.7633 ppm)). ^1^H NMR (400 MHz; DMSO-*d_6_*)) δ (ppm): 0.87 (t, *J* = 6.6 Hz, 3H, aliphatic hydrogens), 1.31 (m, 10H, aliphatic hydrogens), 1.66 (q, 2H, aliphactic hydrogens), 4.11 (t, *J* = 6.6 Hz, 2H, aliphatic hydrogens), 6.57 (d, *J* = 2.0 Hz, 1H, H3), 6.58 (d, *J* = 2.0 Hz, 1H, H4), 7.76 (s, 1H, H2), 8.09 (m, 2H, phen), 8.22 (s, 2H, phen), 8.90 (m, 3H, phen), 9.16 (dd, *J* = 5.2, 1.3 Hz, 1H, phen). ^13^C {^1^H} NMR (100 MHz; DMSO-*d_6_*) δ (ppm): 13.89, 22.02, 25.61, 28.39, 28.60, 28.64 31.18 (aliphatic carbons), 63.13 (C1), 104.92 (C2), 108.55 (C3), 115.86 (C4), 125.74, 125.95, 127.26, 127.46, 129.75, 129.81, 138.84, 138.90, 139.90, 144.24, 145.49, 145.57, 149.59 (phen), 149.71 (C5), 155.56 (C6), 162.62 (C7), 166.78 (C=O). FT-IR spectra in ATR, ν (cm^−1^): 3359, 3077, 3065, 2951, 2923, 2852, 1684, 1600, 1565, 1512, 1491, 1422, 1385, 1336, 1278, 1194, 1152, 1104, 1082, 1053, 988, 965, 898. 836, 762, 745, 722, 708, 602, 565, 507, 485, 431, 342, 309, 301, 268, 241, 227. Λ_M_ (10^−3^ M in DMSO) = 0.52 S cm^2^ mol^−1^.

[Pd(og)(PPh_3_)_2_] **4**

Yield: 89.72%. Color: Grayish purple. Molar Weight (g mol^−1^): 911.3072. Anal. Calcd. for [Pd(C_15_H_20_O_5_)(C_18_H_15_P)_2_]: C, 67.22; H, 5.53%. Found: C, 67.34; H, 5.50 %. HRMS *m*/*z* 911.2262 (Calculated for [Pd(C_15_H_20_O_5_)(C_18_H_15_P)_2_+H]^+^ 911.2241 (Δ +2.3045 ppm)). ^1^H NMR (400 MHz; DMSO-*d_6_*)) δ (ppm): 0.83 (t, *J* = 6.6 Hz, 3H, aliphatic hydrogens), 1.27 (m, 10H, aliphatic hydrogens), 1.57 (q, 2H, aliphactic hydrogens), 4.03 (t, *J* = 6.4 Hz, 2H, aliphatic hydrogens), 5.08 (s, 1H, H2), 6.30 (s, 1H, H3), 6.50 (s, 1H, H4), 7.27–7.37 (m, 12H, PPh_3_), 7.42–7.54 (m, 18H, PPh_3_). ^13^C {^1^H} NMR (100 MHz; DMSO-*d_6_*) δ (ppm): 13.84, 21.96, 25.50, 28.30, 28.52, 31.09 (aliphatic carbons), 63.13 (C1), 102.86 (C2), 109.20 (C3), 115.71 (C4), 127.42, 127.95, 128.27, 128.38, 128.79, 131.23, 131.35, 134.01, 134.13, 134.18, 134.29 (PPh_3_), 143.60 (C5), 154.65 (C6), 163.06 (C–OH), 166.81 (C=O). ^31^P{^1^H} (162 MHz; DMSO-*d_6_*) δ (ppm): 29.68 (d, *J* = 32.4 Hz, P1), 32.36 (d, *J* = 32.4 Hz, P2). FT-IR spectra in ATR, ν (cm^−1^): 3459, 3076, 3052, 2955, 2918, 2853, 1684, 1568, 1494, 1479, 1466, 1435, 1383, 1355, 1341, 1315, 1280, 1198, 1178, 1158, 1097, 1080, 1064, 1025, 998, 968, 866, 825, 766, 759, 750, 739, 707, 689, 617, 592, 542, 525, 510, 494, 459, 423, 404, 369, 339, 297, 281, 250, 238. Λ_M_ (10^−3^ M in DMSO) = 0.14 S cm^2^ mol^−1^.

### 3.2. Biological Studies

#### 3.2.1. Antitubercular Activity

The minimum inhibitory concentration (MIC_90_) of the compounds was determined by Resazurin Microtiter Assay (REMA) [63]. Isoniazid was solubilized in sterile water, while the compounds and rifampicin were solubilized in DMSO; all stock solutions were prepared at 10,000 μg/mL. Compounds and positive control drugs were diluted in Middlebrook 7H9 broth supplemented with 10% oleic acid, albumin, dextrose and catalase (OADC) in a 96-well microplate according to the standardized concentrations (25–0.098 µg/mL). The *Mycobacterium tuberculosis* H37Rv strain was cultivated in Middlebrook 7H9 broth supplemented with 10% OADC at 37 °C until a turbidity equal to that of McFarland standard nº1 was reached; this culture was incubated in the microplate with the compounds diluted in an adjusted concentration of 3 × 10^6^ CFU/mL at 37 °C. On the seventh day, 0.01% resazurin solution (solubilized in sterile water) was added. After 24 h, the fluorescence of the wells was read in a Cytation 3 (Biotek^®^) plate reader (530/590 nm). MIC_90_ was defined as the lowest concentration of the compound capable of inhibiting the growth of 90% of *M. tuberculosis* inoculum. Assays were performed in triplicate, and the results are presented as the mean MIC_90_ values obtained.

#### 3.2.2. Anti-*Campylobacter* Activity

Antibacterial properties were determined with a total of two strains composed of *Campylobacter jejuni* isolated from chicken carcasses by the Ministry of Agriculture, Livestock and Supply of Brazil (MAPA). Strains were chosen that presented mutual resistance to fluoroquinolones (ciprofloxacin MIC ≥ 1 μg/mL) and macrolide (erythromycin MIC = 32 μg/mL) [64]. Samples stored in cryoprotectant enriched with UHT milk were reactivated on *Campylobacter* Agar Base Blood Free (CCDA) (Oxoid^®^) and maintained in microaerophilic (Probac) at 37 °C for 48 h [65]. Typical colonies were then morphologically analyzed for the appearance of curved Gram-negative bacillus on Gram staining. 

#### 3.2.3. *Campylobacter* Count

Antimicrobial susceptibility testing of the strains was performed using serial dilution for subsequent quantification of colonies and verification of logarithmic reduction of planktonic forms. Briefly, the bacterial suspension was standardized to a concentration corresponding to 0.5 on the McFarland scale, and concentrations of 400, 200, 100, 50, 25, 12.5, 6.25, and 3.125 μg/mL of the palladium complex were used. Subsequently, the bacterial suspension was inoculated, and the microplates incubated at 37 °C for 48 h. The reading was characterized by the change in the coloration of the medium and afterwards were quantified at the highest and lowest concentration, respectively. To estimate log_10_ reduction, 100 μL of the highest concentration (400 μg/mL) and 100 μL of the lowest concentration (3.125 μg/mL) were added to sterile saline solution followed by serial dilution for subsequent quantification of colonies and verification of log reduction of planktonic forms. For all tests, negative controls consisting of the medium without added bacteria were used. MIC_90_ and MIC_99_ were defined as the minimum inhibitory concentrations of metal compounds promoting 90% and 99% inhibition, respectively, of the tested bacterial isolates.

#### 3.2.4. Qualitative Evaluation of Biofilm Reduction in *Campylobacter*

The methodology for checking biofilm inhibition described by Kudirkiene et al. (2012) was employed, with modifications [66]. Briefly, 100 μL of the bacterial suspension containing 10^4^ cells prepared in Muller Hinton (MH) broth with 5% *chicken juice* was added into 96-well plates. At the same time, the treatments were combined with the inoculum, followed by complementation of all wells with 100 μL of inoculum to check the inhibition of biomass formation at 400 μg/mL of the palladium compounds and 800 μg/mL peracetic acid (which is the recommended concentration for application in the food industry) [39]. Subsequently, the plates were incubated for 48 h at 37 °C under microaerophilic conditions.

After incubation, the media were removed and dried for 15 min at 55 °C. Total biomass was measured by fixation with 0.1% Crystal Violet (LaborClin®) for 10 min, followed by washing with ultrapure water and then elution with methanol solution. The eluted dye was removed from each well and placed in a new 96-well microtiter plate for reading at OD_600nm_ (biofilm). The assays were performed in eight replicates for three replicates of each strain. For the determination of the Biofilm Formation Index, the following formula was used: BFI = BA − PC/BS, where BFI represents the final result regarding the Biofilm Formation Index, BA the optical density obtained in the mixture of bacteria adhered, PC the absorbance value in the control wells without microorganisms, and BS the optical density (OD_600_) of the suspended cultures in MH with 5% of *chicken juice* [67]. The final classification was as follows: Strong ≥ 1.10; Medium: 0.7–1.10; Weak: 0.35–0.69 and Nonexistent: <0.35.

#### 3.2.5. Scanning Electron Microscopy of Sessile *C. jejuni*

The ultrastructures of the sessile form of the control group, peracetic acid, and metallic compounds were evaluated by Scanning Electron Microscopy (SEM), using a modified method. The inhibition of the formation of biofilms was evaluated in glass spheres with a diameter of 5 mm, in MH in line with the growth conditions described above. After incubation of biomass associated with isolated treatments, the samples were fixed with 2.5% glutaraldehyde and 2.5% paraformaldehyde in 0.1 M PBS buffer (pH 7.4) overnight at 4 °C. Samples were washed three times with PBS buffer. The beads were post-fixed with 1% osmium tetroxide for 1 h and washed three times with PBS buffer. The spheres were dehydrated in a series of ethanol solutions (30, 40, 50, 60, 70, 80, and 90% and then three times at 100%) for 20 min in each step. Samples were dried via CPD (Critical Point Drying) (Leica EM CPD300, Wien, Austria) using liquid carbon dioxide as the transition fluid, then coated with a 20-nm-thick gold layer (SCD 050, Baltec, Germany) and visualized in an MEV VP Zeiss Supra 55 FEG SEM operating at 20 kV.

#### 3.2.6. Statistical Analyses

The results were tabulated and subjected to descriptive statistics with calculation of microbial count reduction expressed in log_10_ for each metal complex. All the assays with the metal complexes were performed in triplicate, and the comparative statistics were determined by applying one-way ANOVA tests. The tests were performed using the Graph Pad Prism 8.0.1 software package, with a 95% confidence interval.

## 4. Conclusions

Herein, four palladium complexes were prepared and fully characterized. The NMR and FTIR data suggest that meg and og act as bidentate ligands coordinating the metal by the oxygen atoms of the meta- and para-positions. With respect to the biological results, three palladium complexes showed good activity against the *M. tuberculosis* H37Rv strain, especially complex **2**, which exhibited an MIC value below 3.28 μg/mL. The antimicrobial results showed promising activity for the control of antibiotic-resistant *C. jejuni*, in particular for complex **1**, which demonstrated very potent activity in comparison to the other compounds evaluated in both life forms. Lastly, the biological effect provided by the complexes containing phenanthroline or phosphine were very different, which denotes a specificity according to the target microorganism and motivate us to carry out new studies with such compounds, which represent a promising alternative for the control of microorganisms of public health importance.

## Figures and Tables

**Table 1:** Anti-MTB activity (MIC) of complexes **1**–**4**, free ligands and control drugs.

Compound	MIC μg/mL
meg	>25
**1**	11.31 ± 0.32
**2**	3.28 ± 0.40
og	22.93 ± 0.46
**3**	>25
**4**	9.06 ± 0.16
Rifampicin	0.10 ± 0.00
Isoniazid	0.23 ± 0.36

## Data Availability

Not applicable.

## Supplementary Materials

The following supporting information can be downloaded at: https://www.mdpi.com/article/10.3390/molecules28093887/s1, Figure S1: ^1^H NMR spectrum of octyl gallate (og); Figure S2: ^1^H NMR spectrum of [Pd(meg)(1,10-phen)] **1**; Figure S3: ^1^H NMR spectrum of [Pd(og)(1,10-phen)] **3**; Figure S4: ^1^H NMR spectrum of [Pd(og)(PPh_3_)_2_] **4**; Figure S5: ^13^C {^1^H} NMR spectrum of methyl gallate (meg).; Figure S6: ^13^C {^1^H} NMR spectrum of octyl gallate (og).; Figure S7: ^13^C {^1^H} NMR spectrum of [Pd(meg)(1,10-phen)] **1**; Figure S8: ^13^C {^1^H} NMR spectrum of [Pd(meg)(PPh_3_)_2_] **2**; Figure S9: ^13^C {^1^H} NMR spectrum of [Pd(og)(1,10-phen)] **3**; Figure S10: ^13^C {1H} NMR spectrum of [Pd(og)(PPh_3_)_2_] **4**; Figure S11: ^31^P{^1^H} NMR spectrum of [Pd(meg)(PPh_3_)_2_] **2**; Figure S12: ^31^P{^1^H} NMR spectrum of [Pd(og)(PPh_3_)_2_] **4**; Figure S13: FTIR spectrum of [Pd(meg)(1,10-phen)].2H_2_O **1**; Figure S14: FTIR spectrum of [Pd(meg)(PPh_3_)_2_] **2**; Figure S15: FTIR spectrum of [Pd(og)(1,10-phen)].2.5H_2_O **3**; Figure S16: FTIR spectrum of [Pd(og)(PPh_3_)_2_] **4**; Figure S17: TGA/DTA curve of complex **1**.
