# Supplementary material for: New Palladium(II) Complexes Containing Methyl Gallate and Octyl Gallate: Effect against Mycobacterium tuberculosis and Campylobacter jejuni
Source: Molecules. 2023 May 5;28(9):3887. doi: 10.3390/molecules28093887 (PMC10179749; doi:10.3390/molecules28093887)
Supplement: Supplementary file 1 [file molecules-28-03887-s001.zip › molecules-2253734-supplementary.pdf]

Figure S1. ^{1}H NMR spectrum of octyl gallate (og).

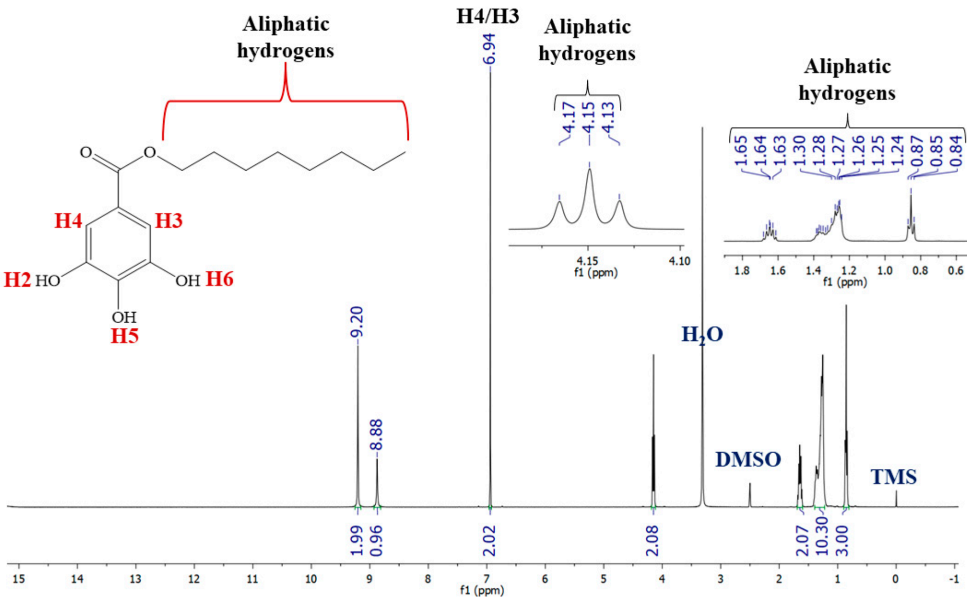

Figure S2. ^{1}H NMR spectrum of [Pd(meg)(1,10-phen)] **1**.

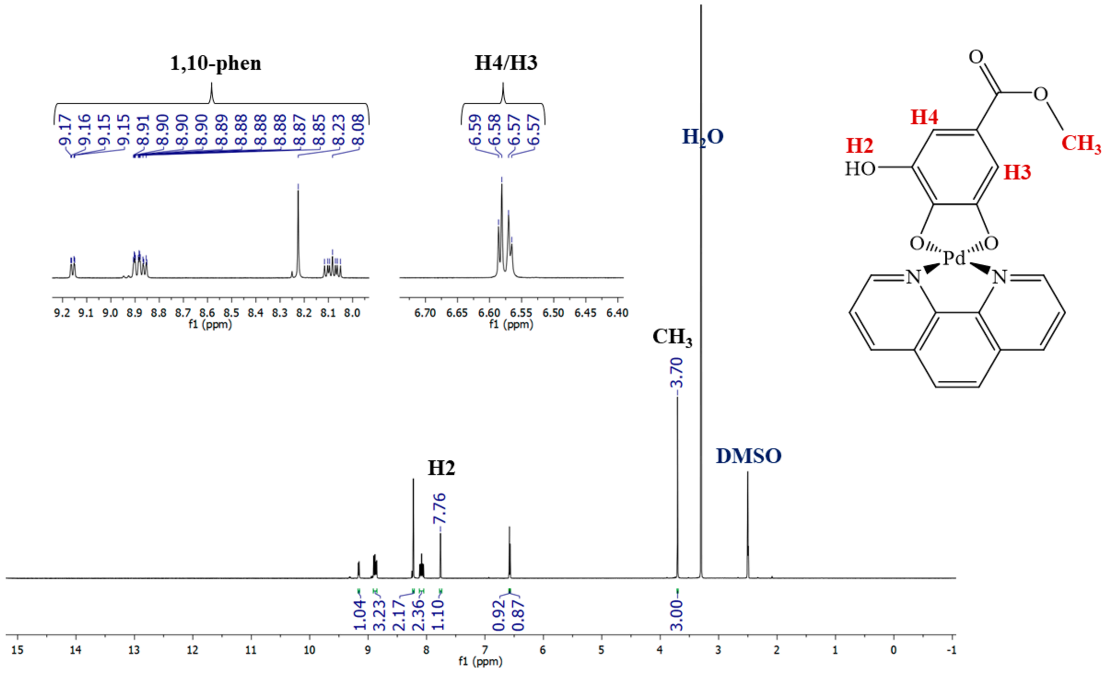

Figure S3. ^{1}H NMR spectrum of [Pd(og)(1,10-phen)] **3**.

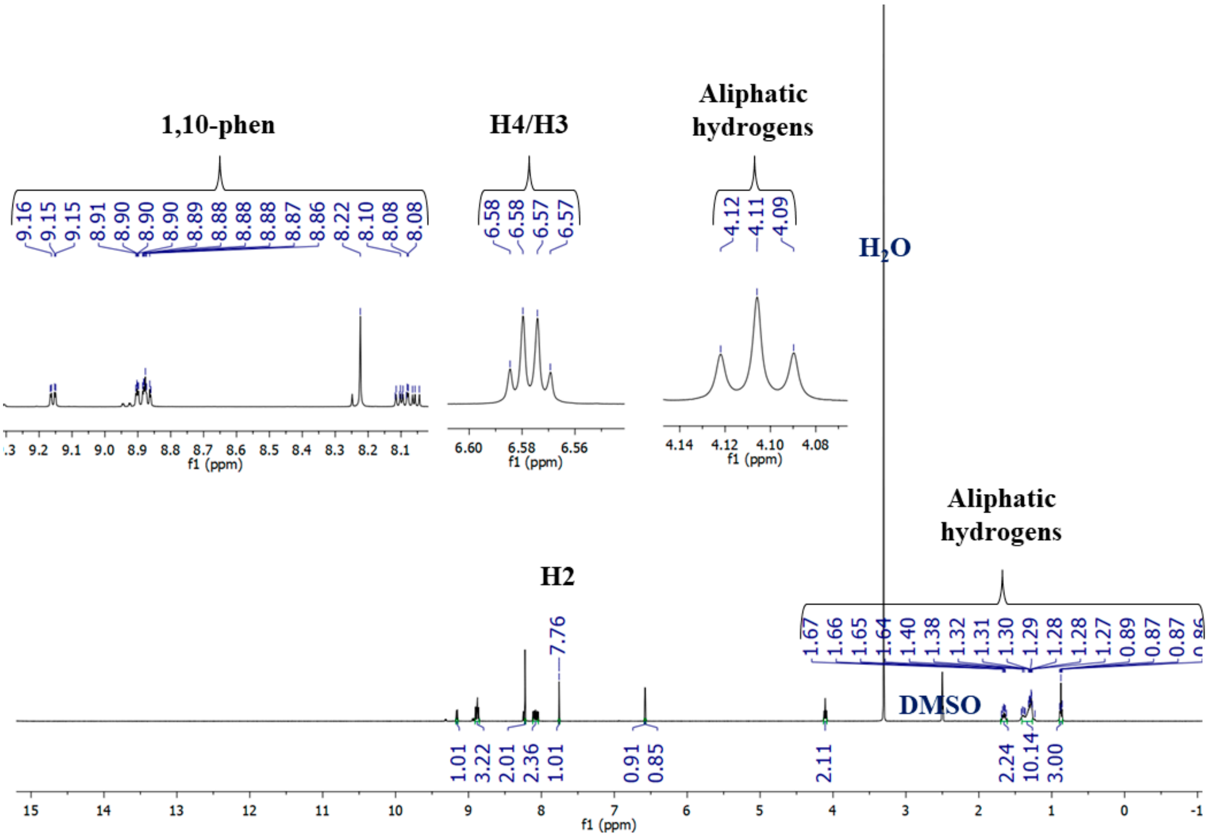

Figure S4. ^{1}H NMR spectrum of [Pd(og)(PPh3)2] **4**.

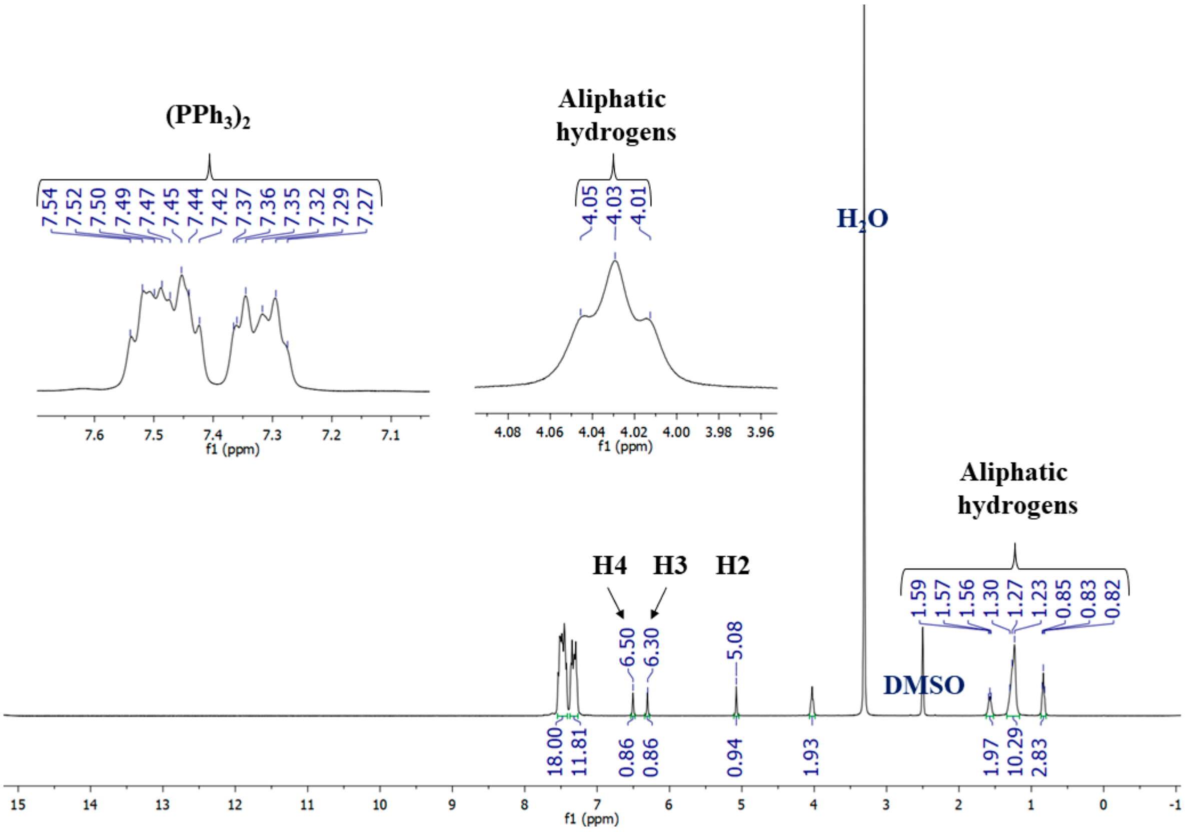

Figure S5. ^{13}C {^{1}H} NMR spectrum of methyl gallate (meg).

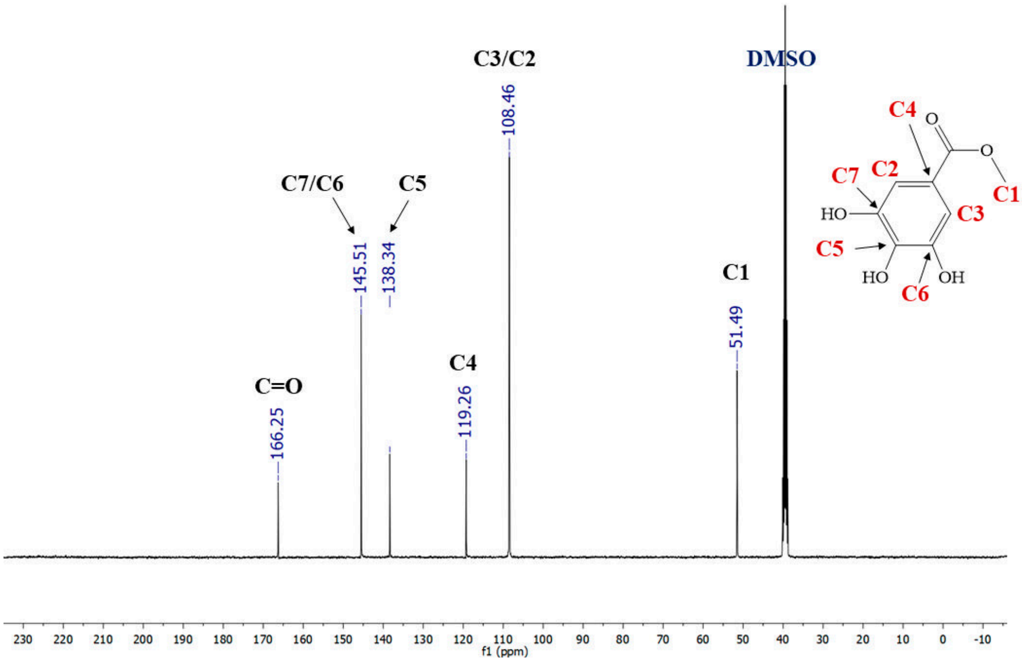

Figure S6. ^{13}C {^{1}H} NMR spectrum of octyl gallate (og).

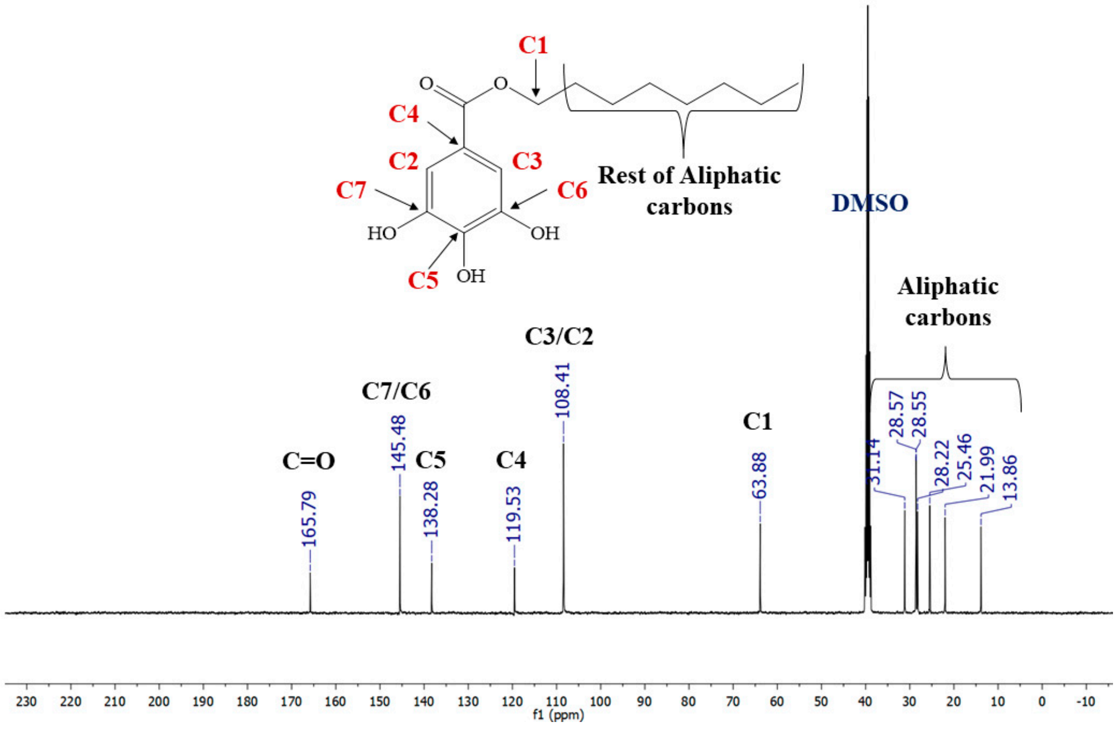

Figure S7. ^{13}C {^{1}H} NMR spectrum of [Pd(meg)(1,10-phen)] **1**.

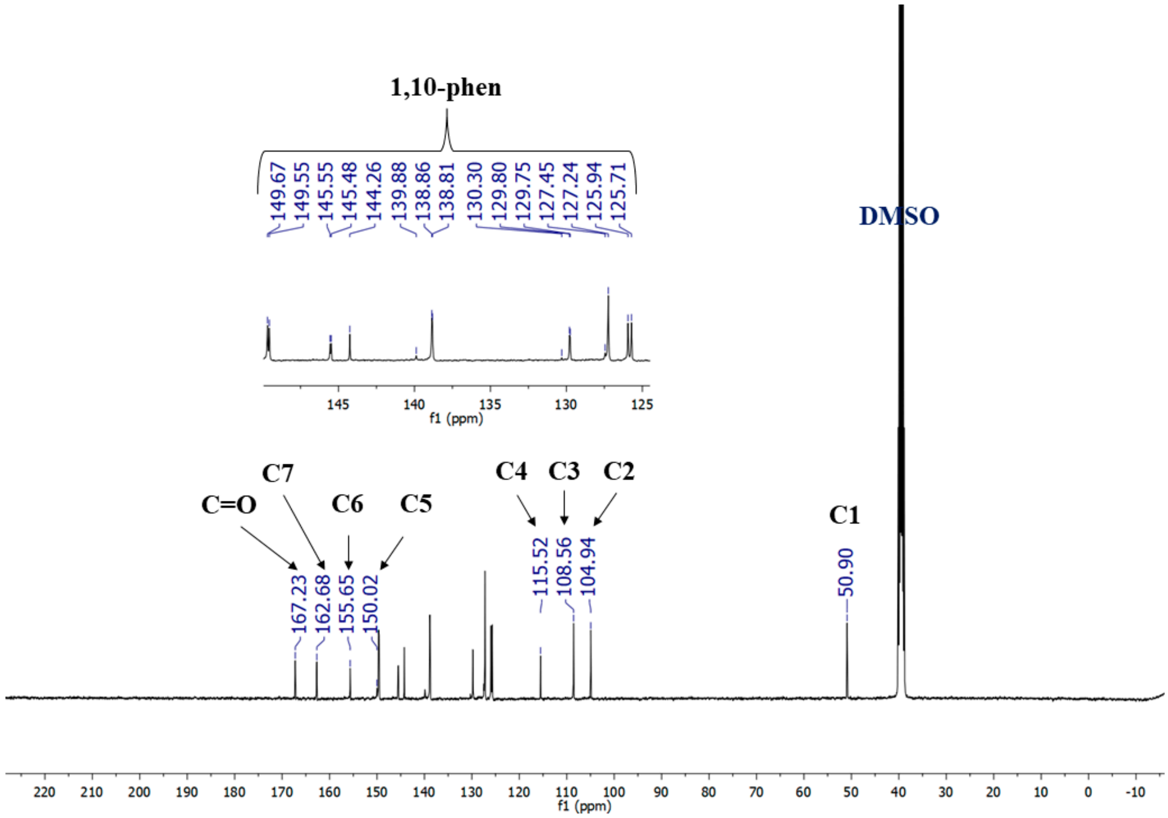

Figure S8. ^{13}C {^{1}H} NMR spectrum of [Pd(meg)(PPh_3)$_2$] **2**.

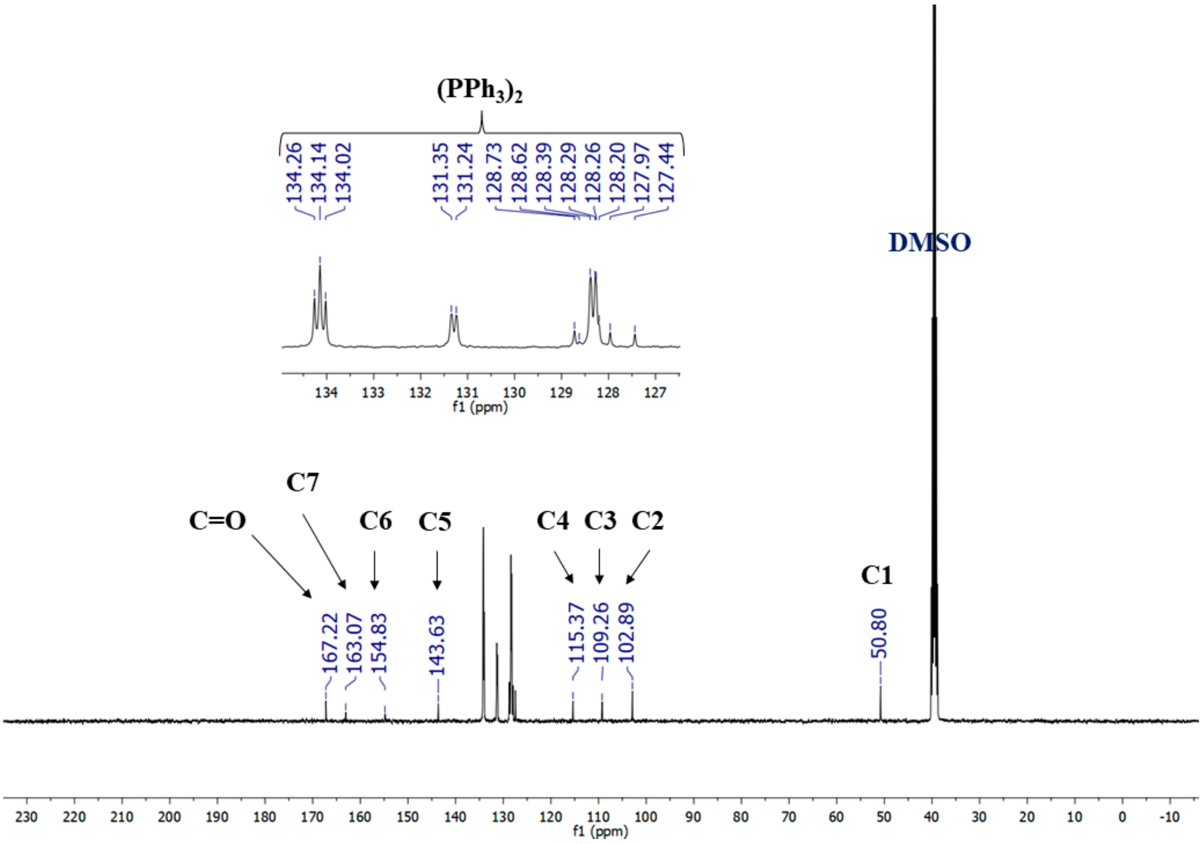

Figure S9. ^{13}C {^{1}H} NMR spectrum of [Pd(og)(1,10-phen)] **3**.

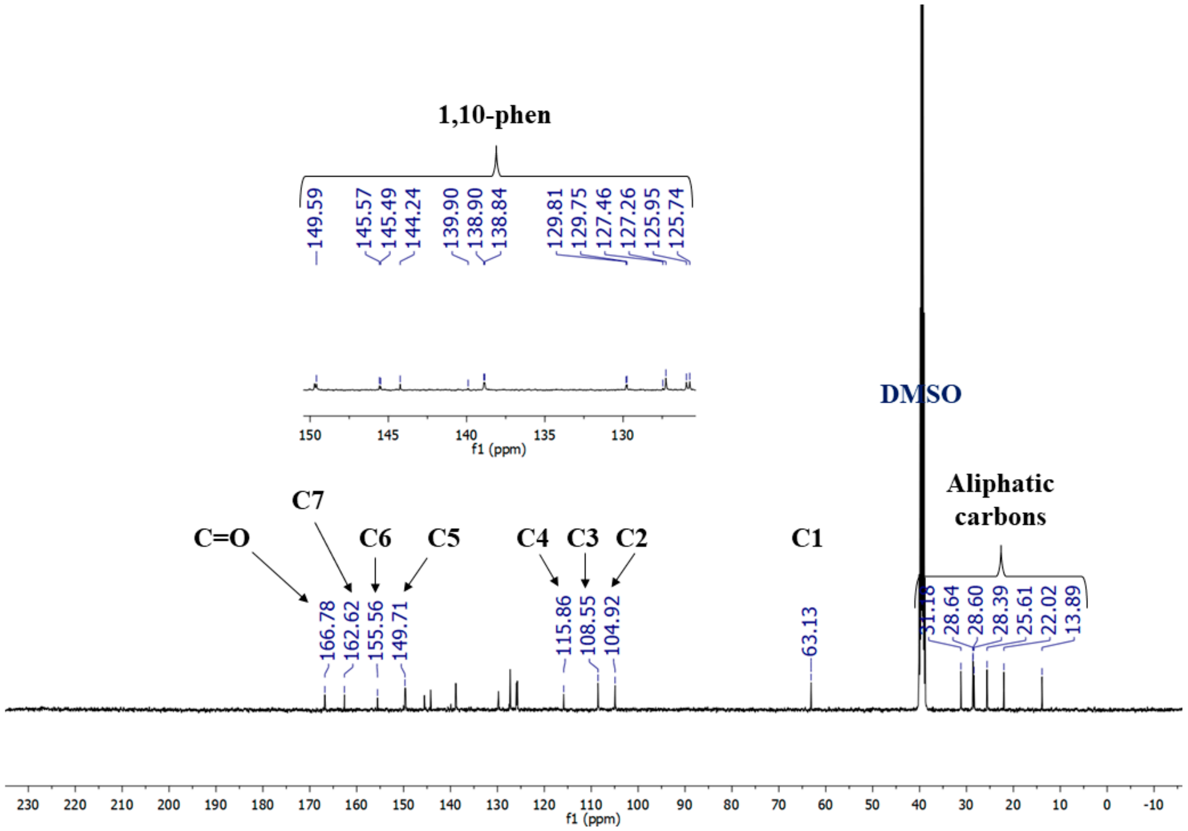

Figure S10. ^{13}C {^{1}H} NMR spectrum of $[Pd(og)(PPh_3)_2]$ **4**.

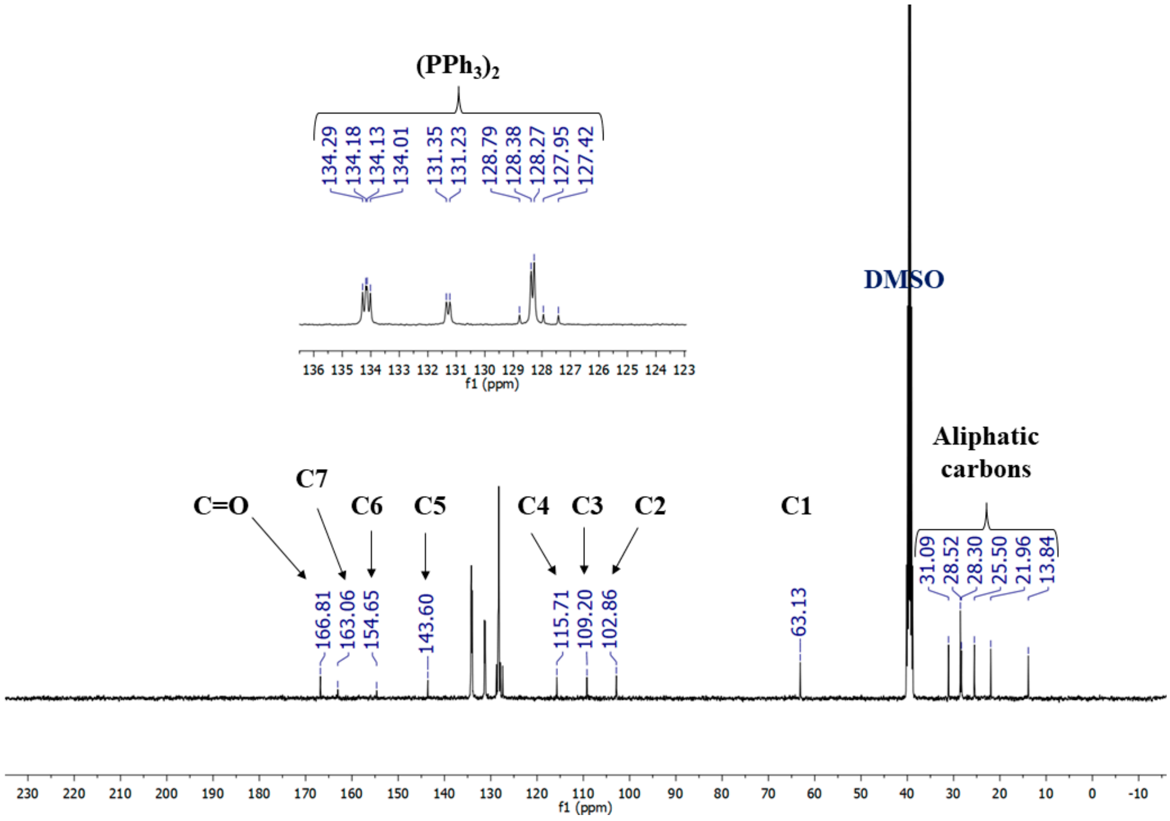

Figure S11. $^{31}P\{^1H\}$ NMR spectrum of $[Pd(meg)(PPh_3)_2]$ **2.**

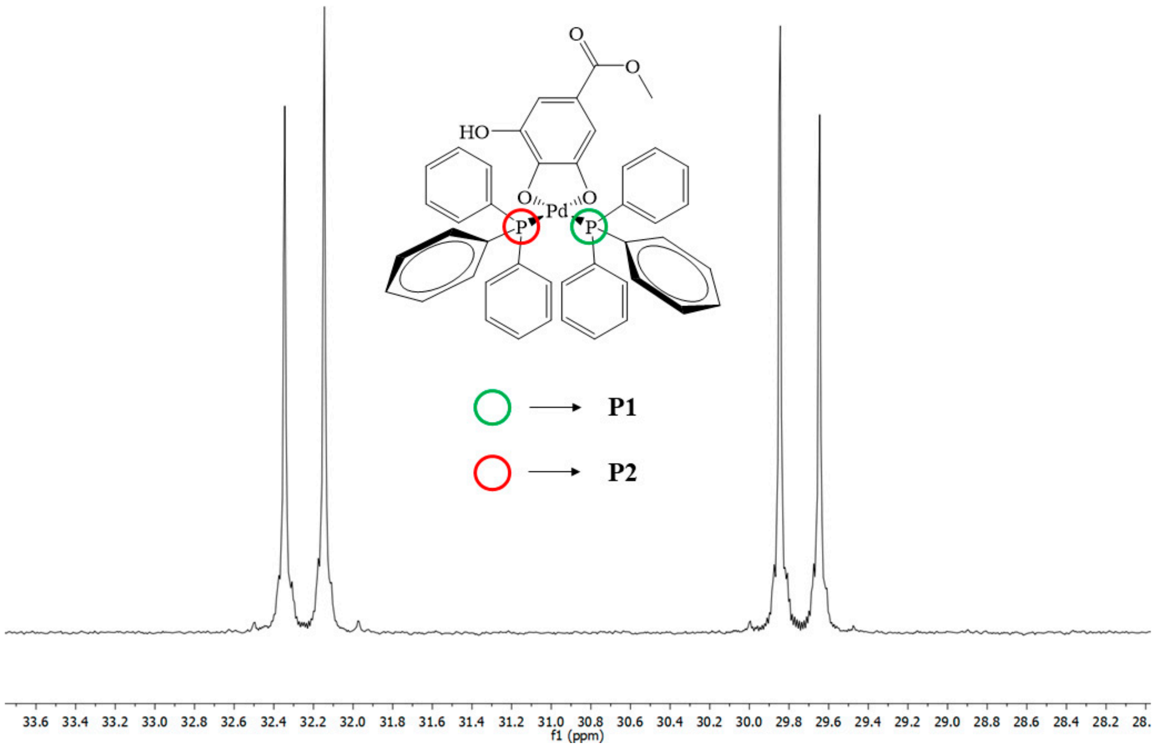

Figure S12. $^{31}P\{^1H\}$ NMR spectrum of $[Pd(og)(PPh_3)_2]$ **4**.

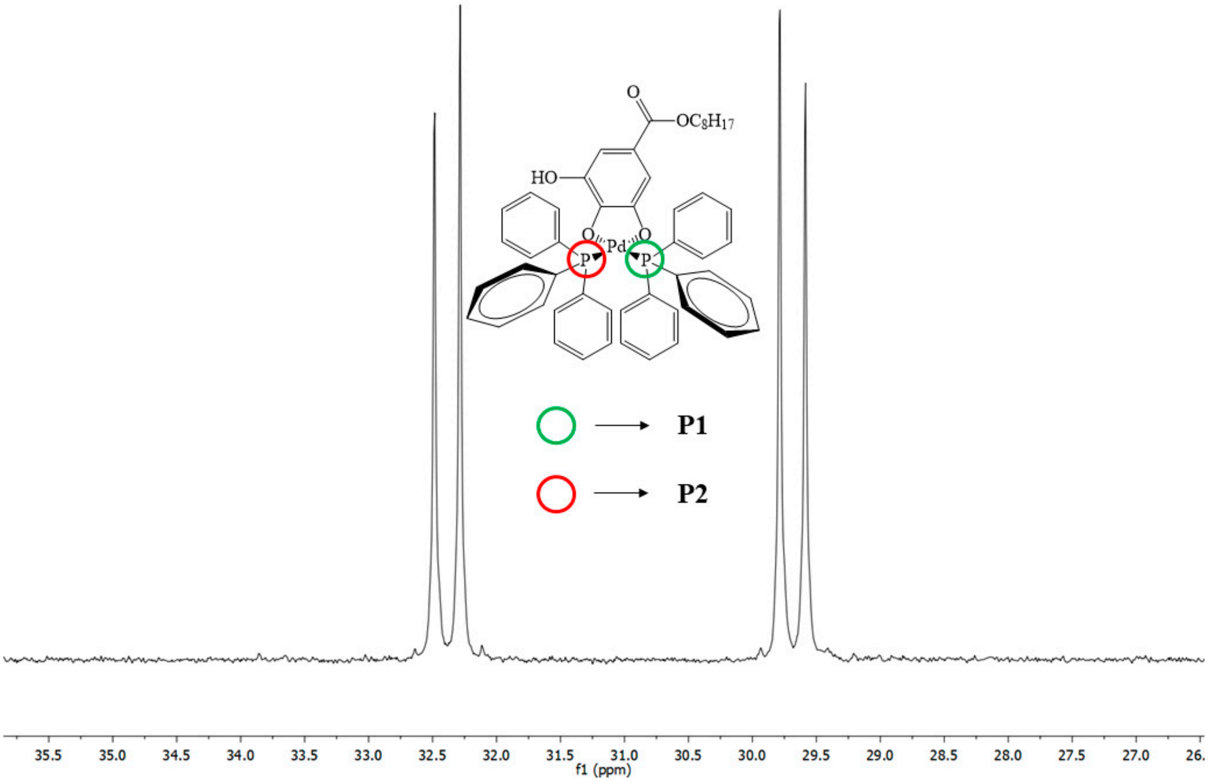

Figure S13. FTIR spectrum of [Pd(meg)(1,10-phen)].2H2O **1**.

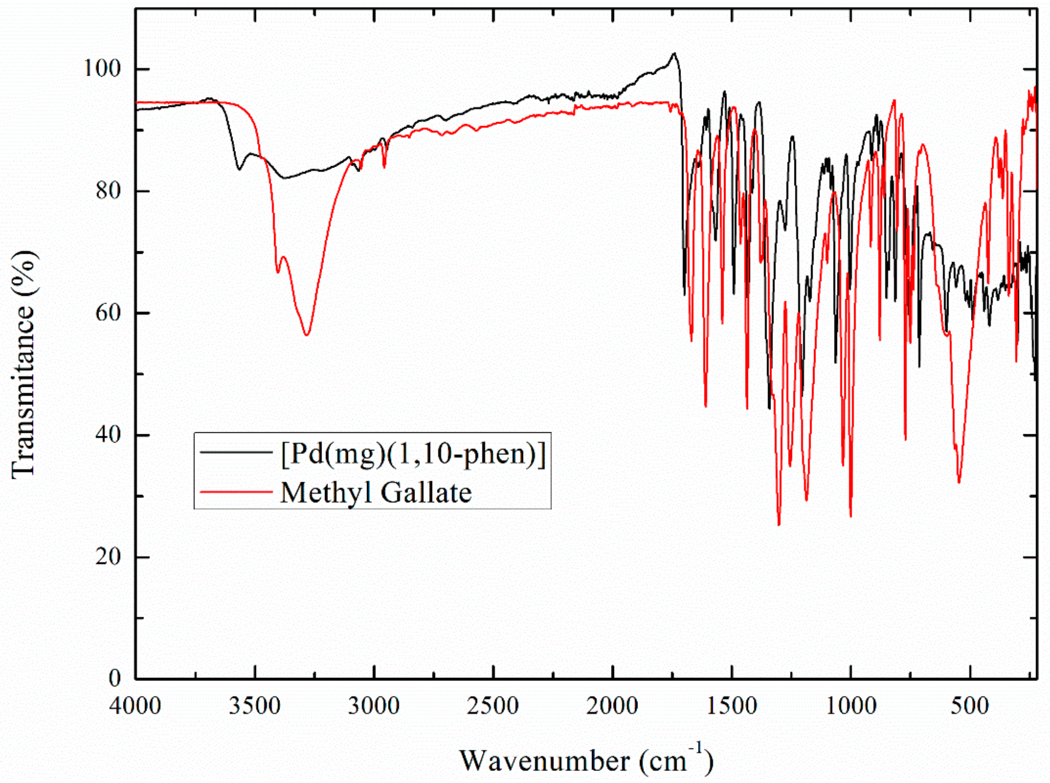

Figure S14. FTIR spectrum of $[Pd(meg)(PPh_3)_2]$ **2**.

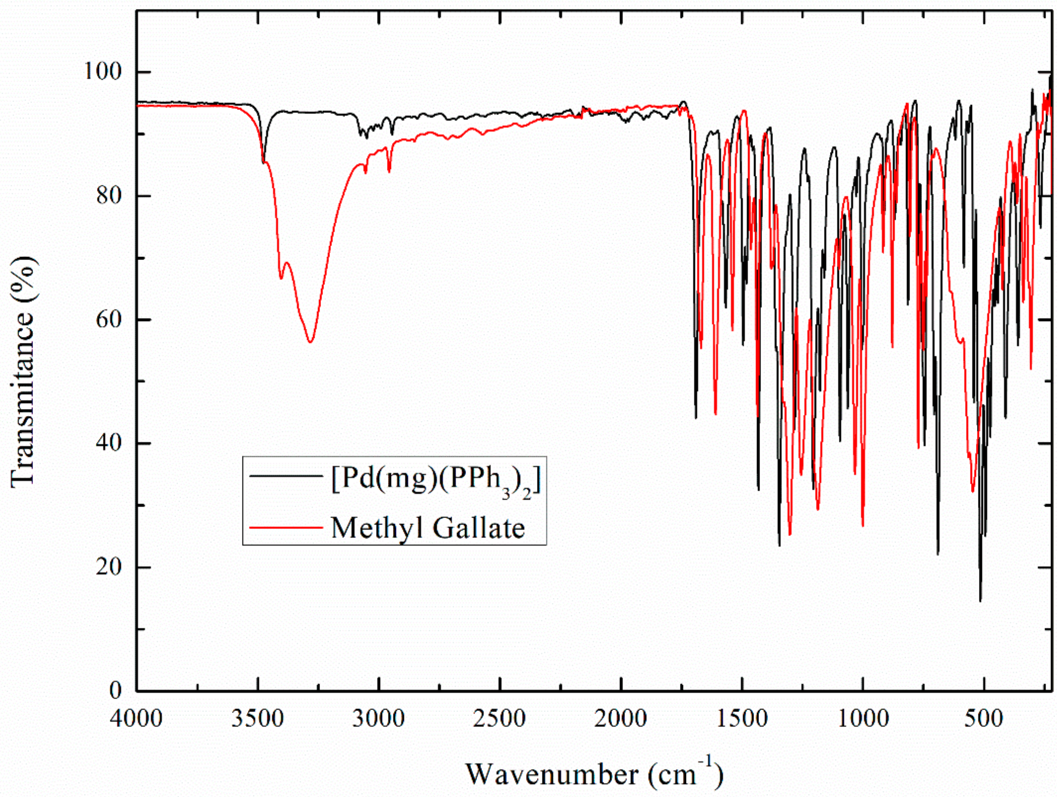

Transmitance (%)
Wavenumber (cm^{-1})
[Pd(mg)(PPh$_3$)$_2$]
Methyl Gallate
0
20
40
60
80
100
4000
3500
3000
2500
2000
1500
1000
500

Figure S15. FTIR spectrum of[Pd(og)(1,10-phen)].2.5H_2O **3**.

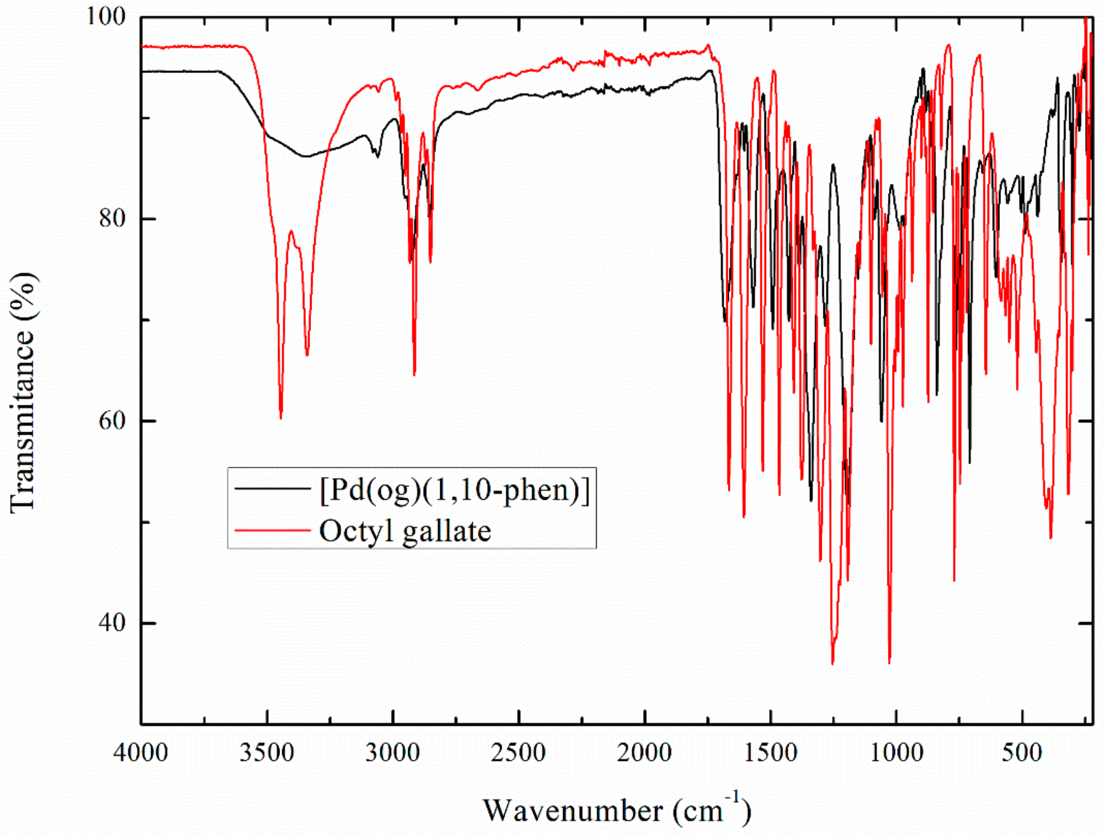

Figure S16. FTIR spectrum of [Pd(og)(PPh_3)$_2$] **4**.

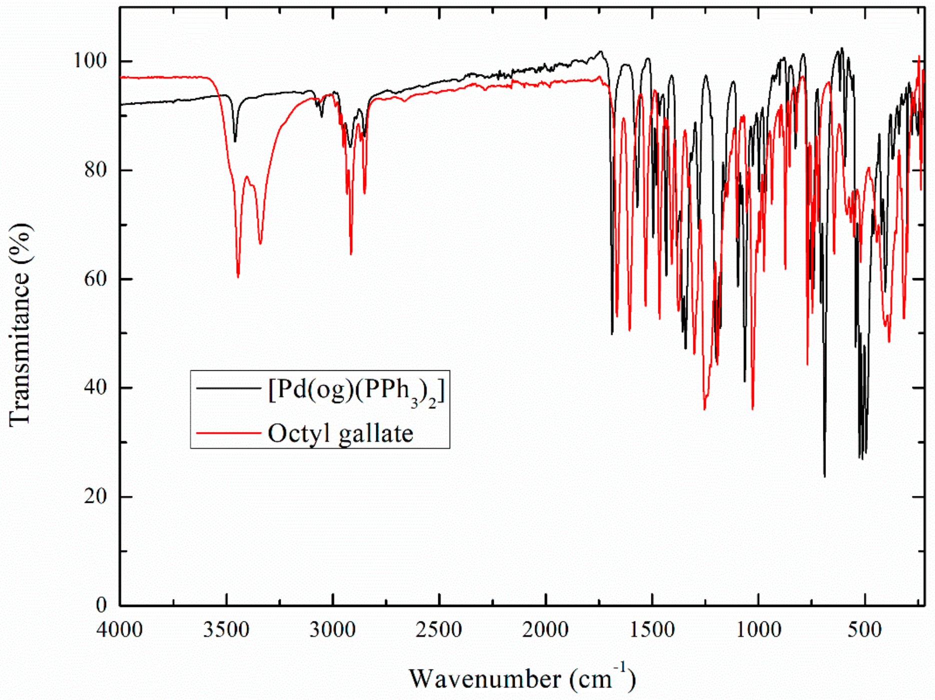

Figure S17. TGA/DTA curve of complex **1**.

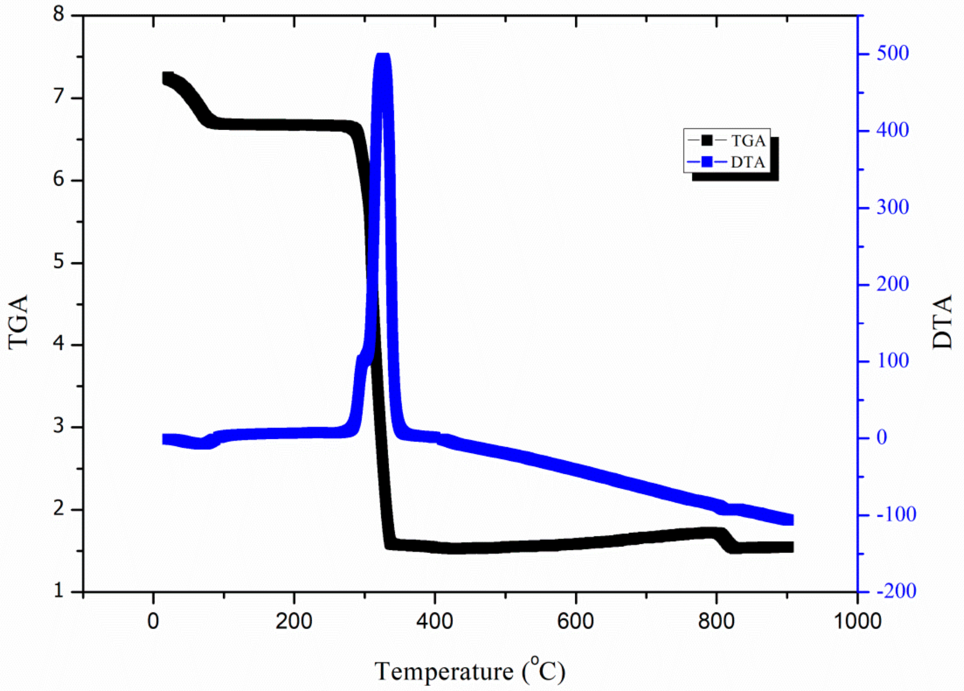

TGA
DTA
Temperature (°C)
0
200
400
600
800
1000
1
2
3
4
5
6
7
8
-200
-100
0
100
200
300
400
500
